# Systematic Review of the Ovitrap Surveillance of *Aedes* Mosquitoes in Brazil (2012–2022)

**DOI:** 10.3390/tropicalmed10080212

**Published:** 2025-07-28

**Authors:** Raquel Fernandes Silva Chagas do Nascimento, Alexandre da Silva Xavier, Tania Ayllón Santiago, Daniel Cardoso Portela Câmara, Izabel Cristina dos Reis, Edson Delatorre, Patrícia Carvalho de Sequeira, Vitor Henrique Ferreira-de-Lima, Tamara Nunes Lima-Camara, Nildimar Alves Honório

**Affiliations:** 1 Laboratório das Interações Vírus Hospedeiros-LIVH, Instituto Oswaldo Cruz, Fundação Oswaldo Cruz, Rio de Janeiro 21040-360, Brazil; 2 Núcleo Operacional Sentinela de Mosquitos Vetores-Nosmove/Fiocruz, Fundação Oswaldo Cruz, Rio de Janeiro 21040-360, Brazil; 3 Laboratório de Mosquitos Transmissores de Hematozoários-LATHEMA, Instituto Oswaldo Cruz, Fundação Oswaldo Cruz, Rio de Janeiro 21040-360, Brazil; 4 Departamento de Sanidad Animal, Facultad de Veterinaria, Universidad Complutense de Madrid, 28040 Madrid, Spain; 5 Centro de Vigilancia Sanitaria Veterinaria (VISAVET), Universidad Complutense de Madrid, 28040 Madrid, Spain; 6 Programa de Computação Científica-PROCC/Fiocruz, Fundação Oswaldo Cruz, Rio de Janeiro 21040-360, Brazil; 7 Instituto de Comunicação e Informação Científica e Tecnológica em Saúde-ICICT/Fiocruz, Fundação Oswaldo Cruz, Rio de Janeiro 21040-360, Brazil; 8 Laboratório de Genômica e Ecologia Viral, Centro de Ciências da Saúde, Universidade Federal do Espírito Santo, Vitória 29075-910, Brazil; 9 Laboratório de Arbovírus e Vírus Hemorrágicos, Instituto Oswaldo Cruz, Fundação Oswaldo Cruz, Rio de Janeiro 21040-360, Brazil; 10 Departamento de Epidemiologia, Faculdade de Saúde Pública, Universidade de São Paulo, São Paulo 01246-904, Brazil

**Keywords:** *Aedes aegypti*, *Aedes albopictus*, arboviruses, ovitraps, surveillance, Brazil

## Abstract

**Background:** Arthropod-borne diseases primarily affect tropical and subtropical regions, exhibiting seasonal patterns that peak during hot and rainy months when conditions favor mosquito vector proliferation. Factors such as high temperatures, elevated humidity, rainfall, urbanization, and the abundance of natural and artificial breeding sites influence *Aedes* vector dynamics. In this context, arboviruses pose significant public health challenges, likely worsened by global warming. In Brazil, *Aedes* (*Stegomyia*) *aegypti* (Linnaeus, 1762) is the primary vector for yellow fever, dengue, chikungunya, and Zika. *Aedes* (*Stegomyia*) *albopictus* (Skuse, 1894) is an important global arbovirus vector and is considered a potential vector in Brazil. Entomological surveillance of these species often uses oviposition traps targeting immature stages. Evaluating studies that use ovitraps to collect *Ae. aegypti* and *Ae. albopictus* egg is essential for improving mosquito surveillance strategies. This study systematically reviewed peer-reviewed articles on ovitrap-based surveillance of *Aedes* mosquitoes in Brazil, published in Portuguese and English from 2012 to 2022. The findings suggest that ovitraps are an effective method for detecting the presence or absence of *Ae. aegypti* and *Ae. albopictus*, serving as a reliable proxy for estimating mosquito abundance in Brazilian contexts.

## 1. Introduction

Arthropod-borne viruses (arboviruses), including yellow fever, dengue, Zika, and chikungunya, are transmitted by hematophagous mosquitoes and pose a major public health challenge in tropical and subtropical regions, including Brazil [1,2,3]. The primary vectors of these arboviruses to humans are the mosquito species *Aedes (Stegomyia) aegypti* (Linnaeus, 1762) and *Aedes (Stegomyia) albopictus* (Skuse, 1894) (Diptera: Culicidae) [4]. During the transmission cycle, female mosquitoes acquire the virus through a blood meal from an infected vertebrate host and, following viral replication, can transmit the pathogen to another host [1,5,6]. Arbovirus transmission in *Ae. aegypti* and *Ae. albopictus* occurs via horizontal or vertical pathways. Horizontal transmission involves virus acquisition through viremic vertebrate hosts. Blood feeding is associated with the hematophagous behavior of females needed for egg maturation, and venereal transmission entails sexual transfer of the virus between infected and uninfected partners [6,7,8,9,10]. Vertical transmission, in which the virus is passed from infected females to their offspring, is critical for arbovirus persistence [5,10,11]. It can occur through three modes: transovarial transmission (infection of germ cells before chorion formation), transovum transmission (infection during ovulation of chorionated eggs), and oocyte surface contamination (adherence of the virus to the chorion, remaining through hatching and infecting the emerging larvae) [5].

The primary arbovirus vector, the urban and semi-urban *Ae. aegypti*, was introduced into Brazil during the 18th and 19th centuries, likely as a result of the African slave trade [4]. Now widespread across the country, *Ae. aegypti* is closely associated with human populations because of its anthropophilic behavior and preference for laying eggs in artificial containers, such as abandoned tires, plastic buckets, glass bottles, and dish plates [4,12,13,14]. This species disperses rapidly and favors oviposition in residential and peridomestic areas, where females locate standing water and lay eggs just below the surface [1,15,16,17,18]. If the water evaporates, the eggs can remain viable for up to a year [19], reactivating upon renewed contact with water. Adult mosquitoes are most active during early morning and dusk, coinciding with peak host-seeking activity for blood meals [4,20].

The potential arbovirus vector *Ae. albopictus* is considered the most invasive mosquito species worldwide because of its adaptability and efficient dispersal capacity [15,21]. First recorded in Brazil in 1986 in the state of Rio de Janeiro, this species was likely introduced via commercial trade routes from one or more Asian countries [22,23,24]. Despite its potential role in dengue virus transmission, *Ae. albopictus* remains underprioritized in Brazil’s epidemiological surveillance strategies [3,25]. Nevertheless, this species should not be overlooked, especially given recent studies demonstrating its susceptibility to infection and ability to transmit dengue and chikungunya viruses through vector competence assays [25,26,27]. *Aedes aegypti* and *Ae. albopictus* are sympatric species capable of coexisting in the same environment, often exploiting distinct breeding sites shaped by human activity, particularly in peridomestic and transitional urban–rural settings [4,15,22].

Entomological surveillance of *Ae. aegypti* and *Ae. albopictus* involves the use of various vector monitoring strategies [28]. Different methods are applied to detect both immature and adult stages of these *Aedes* species. Therefore, employing a highly sensitive approach is essential for the success of surveillance programs targeting these vectors [29,30,31]. One of the most sensitive and widely adopted methods is the oviposition trap, commonly referred to as an ovitrap [32]. First described in 1965 and improved in 1966 [33], the ovitrap typically consists of wooden paddles used as oviposition substrates and has been extensively employed to detect mosquito populations in specific areas [29,30,32]. Ovitraps have been recognized as valuable surveillance tools [34], offering a low-cost means to gather detailed data on mosquito population dynamics [31,35]. They are also used to monitor the geographic distribution, density, frequency, and seasonal variation of mosquito populations [29,36,37]. By quantifying collected eggs, ovitraps contribute significantly to detecting the presence or absence of *Ae. aegypti* and *Ae. albopictus* [28,38]. Additionally, they aid in identifying arbovirus risk factors using *Aedes* egg density indices [39,40] and support the development of efficient surveillance systems. Although ovitraps do not directly assess adult mosquito populations, they effectively capture population trends and fluctuations [31].

In Brazil, ovitraps have been evaluated in multicenter entomological surveys across various municipalities and compared to other mosquito trapping methods [31,41]. Additionally, the Brazilian Ministry of Health has published several documents on ovitrap implementation and recently included ovitraps among its recommended strategies for entomological surveillance in the updated Guidelines for the Prevention and Control of Urban Arboviruses, advocating their use nationwide [42,43]. In this context, the present study is a systematic review of publications reporting Brazilian research that employed ovitraps to monitor *Aedes* populations, specifically *Ae. aegypti* and *Ae. albopictus* published from 2012 to 2022. The primary objective is to synthesize surveillance data obtained through this oviposition-based method.

## 2. Materials and Methods

This systematic review followed the PICO strategy (Population, Intervention, Comparison, and Outcome) [44]. Inclusion criteria encompassed peer-reviewed scientific articles published in open-access journals from 2012 to 2022 that reported studies using ovitraps for *Aedes* monitoring in Brazil. Dissertations, theses, conference presentations, websites, manuals, booklets, and other non-peer-reviewed sources were excluded. The review included publications in Portuguese or English that specifically addressed *Ae. aegypti* and *Ae. albopictus*.

**Figure 1:**
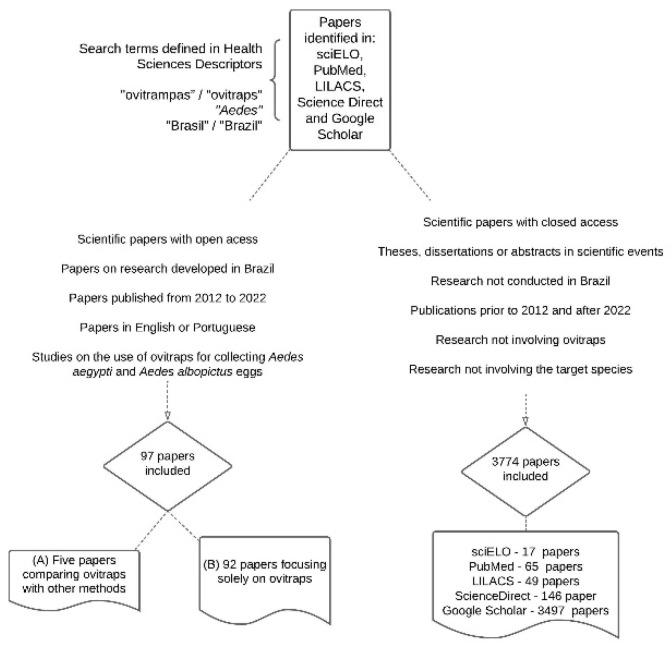
Schematic overview of procedures adopted in the systematic literature review reported.

Literature searches were conducted across five indexed databases and web search engines: Scientific Electronic Library Online–SciELO (http://www.scielo.org), PubMed-Medline (https://pubmed.ncbi.nlm.nih.gov/), LILACS (https://lilacs.bvsalud.org), Science Direct (https://www.sciencedirect.com/), and Google Scholar (https://scholar.google.com.br/?hl=pt). These platforms were accessed at different times during the years 2023 and 2024. Two search profiles were used to identify ovitrap studies (Figure 1): one with Portuguese terms and one with their English equivalents. The Portuguese terms included “ovitrampas,” “*Aedes*,” and “Brasil,” while the English terms were “ovitraps,” “*Aedes*,” and “Brazil.” All terms were combined using the Boolean operator “AND.” The terms “ovitrampas” and “ovitraps” were the only ones not indexed in the Health Sciences Descriptors (DeCS) vocabulary [45].

### Data Analysis

Descriptive analyses were conducted using R version 4.4.3 [46]. The dataset was summarized using frequencies and proportions, and visualizations were created to illustrate the distribution of publications over time, study durations, regional geographic coverage, and temporal trends in entomological indicators. Mean values of the Ovitrap Positivity Index (OPI) and Egg Density Index (EDI) were manually extracted from studies that reported both indicators and were included in the trend analysis. For the temporal smoothing trends of the entomological indicators, a midpoint year was calculated for each study by averaging the reported start and end years of data collection. These midpoint values were then used as a temporal reference for plotting OPI values over time. Smoothing trends for entomological indicators were modeled using the LOESS non-parametric regression method [47]. Data visualizations were produced using the ggplot2 package and related extensions [48]. Spatial analyses were based on official Brazilian geographic boundaries from the geobr package [49].

## 3. Results

A total of 3871 articles were initially identified. After applying the inclusion and exclusion criteria, 698 papers in Portuguese and 3076 in English were excluded. Ultimately, a total of 97 articles were selected across the five search platforms, including seventy-five published in English, nineteen articles in Portuguese, and three in both languages.

**Figure 2:**
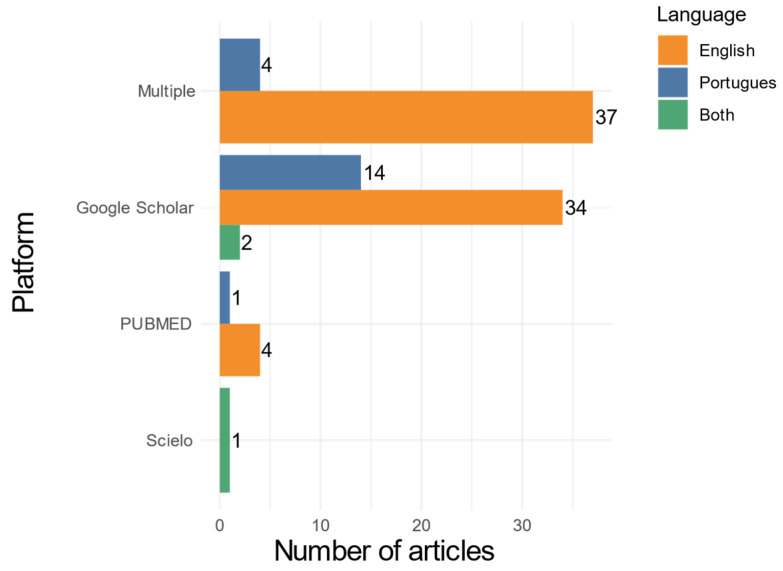
Number of articles published in Portuguese, English, and in both languages across Google Scholar, PubMed, ScienceDirect, LILACS and SciELO platforms, during the period 2012–2022.

An analysis of the distribution of articles by language and indexing platform revealed a predominance of English-language publications, with broad dissemination across international databases. Among the seventy-five English-language articles, thirty-seven were indexed on multiple platforms, thirty-four were retrieved exclusively from Google Scholar, and four were found in PubMed. Of the nineteen Portuguese-language articles, fourteen were available on Google Scholar, four were indexed on multiple platforms, and one was found in PubMed. Additionally, two bilingual articles (Portuguese/English) were identified in Google Scholar, and one was indexed in SciELO. These findings suggest some overlap across platforms, particularly for English-language publications, indicating greater visibility and accessibility through widely used indexing services. The distribution of articles by language and indexing platform is illustrated in Figure 2.

**Figure 3:**
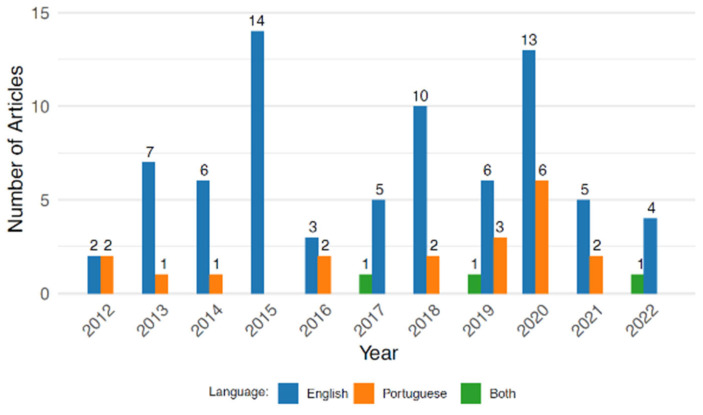
Number of articles related to ovitrap studies published by year and by language (Portuguese, English, and both), from 2012 to 2022.

The number of articles published on ovitraps by year is presented in Figure 3. Annual publication counts ranged from one to fourteen, with a predominance of English-language publications throughout the study period. Notable peaks occurred in 2015 (14 articles), 2018 (10 articles), and 2020 (13 articles). In contrast, Portuguese-language publications were fewer and more sporadic, with the highest count in 2020 (six articles). Bilingual publications (Portuguese and English in the same article) were rare and identified only in 2017, 2019, and 2022.

**Figure 4:**
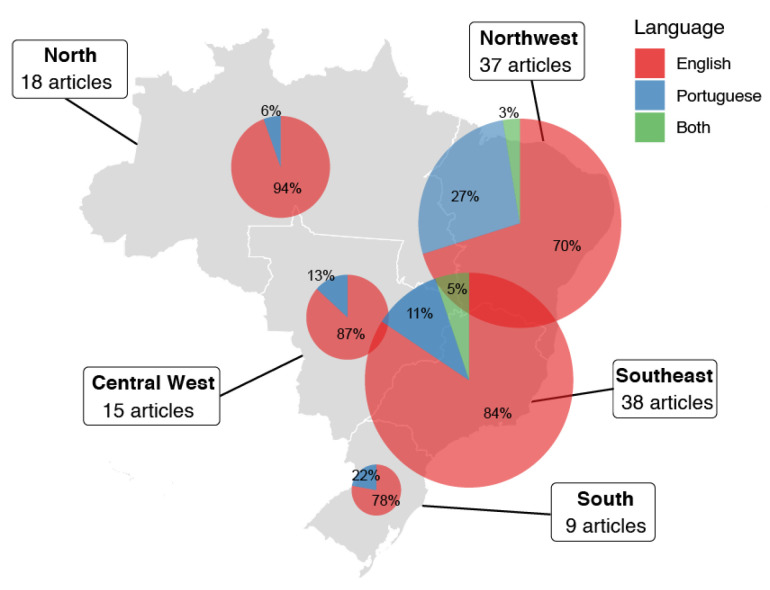
Number of articles related to ovitrap studies published by the Brazilian region and by language (Portuguese, English, and both), from 2012 to 2022. Articles addressing more than one region were counted separately for each region covered.

Figure 4 shows the regional distribution of the scientific articles identified in this review, categorized by language. The southeast and northeast regions accounted for the highest number of publications, with 38 and 37 articles, respectively. In the southeast, 84.2% of the articles were in English, 10.5% in Portuguese, and 5.3% were bilingual. The northeast presented a predominance of Portuguese-language publications (73.0%), followed by English (18.9%) and bilingual (8.1%). In the north (18 articles), 94.4% were published in English and 5.6% in Portuguese, with no bilingual publications. The central–west (15 articles) and south (9 articles) also showed a predominance of English-language publications (86.7% and 77.8%, respectively). Portuguese-language articles in these regions were limited (13.3% in the central–west and 22.2% in the south), with no bilingual publications reported.

**Figure 5:**
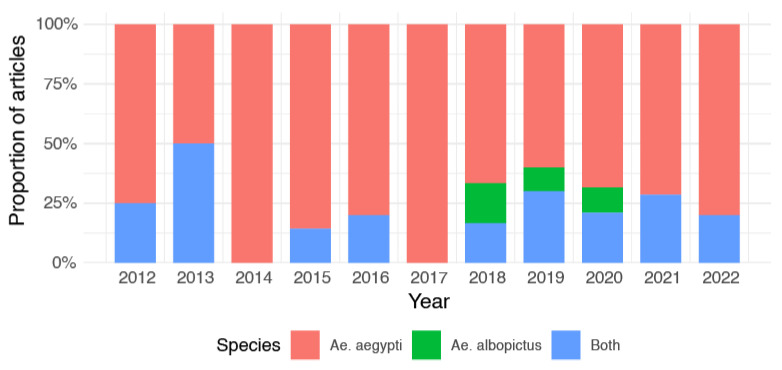
Proportion of articles related to ovitrap studies published by species (*Ae. aegypti*, *Ae. albopictus*, and both) by year, from 2012 to 2022.

The articles identified through this review were first organized chronologically (Supplementary Tables S1 and S2), and then categorized by *Aedes* species and language. Table S1 presents a total of 24 articles (24.75%) focused on *Aedes aegypti* (16), *Aedes albopictus* (0), or both species (8), containing keywords in Portuguese or both Portuguese and English. Table S2 includes 78 articles (80.41%) targeting *Ae. aegypti* (59), *Ae. albopictus* (5), or both species (14), with keywords in English or both Portuguese and English. Both tables are available in the Supplementary Materials. The number of articles published by *Aedes* species over time is shown in Figure 5. The temporal distribution of articles focusing on *Ae. aegypti* (72 articles; 74.22%) was higher than those focusing exclusively on *Ae. albopictus* (5 articles; 5.15%) (Figure 5). Additionally, 21 articles (21.6%) investigated both species, reflecting a more integrated entomological approach in certain studies. The number of studies on *Ae. aegypti* studies increased steadily from 2012, peaking in 2015 (12 articles) and again in 2020 (13 articles), possibly in response to arboviral outbreaks and intensified surveillance. Although studies including *Ae. albopictus* (alone or with *Ae. aegypti*) have grown modestly in recent years, their overall volume remains limited, underscoring the need for broader entomological monitoring of this potential but epidemiologically important vector.

In this systematic review, we identified various types of ovitraps used for *Aedes* mosquito surveillance. A total of fifty-nine were traditional ovitraps containing either water or water with hay infusion, five combined ovitraps with larvicides, twenty-seven incorporated plant extracts, one used water with cat food, and five utilized brewer’s yeast.

**Figure 6:**
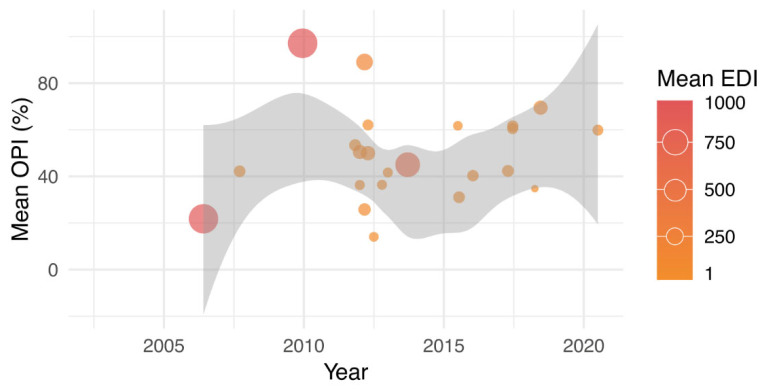
Temporal dynamics of the mean ovitrap positivity index (OPI) and its relationship with the mean egg density index (EDI) across 23 studies. The green line represents the OPI trend; the size and color of the circles indicate the average EDI.

Regarding entomological indicators, 46 studies used qualitative indices such as the ovitrap positivity index (OPI), while 66 studies employed quantitative indices, including the number of *Aedes* eggs, Mean Egg Index, or Egg Density Index (EDI). Figure 6 illustrates the temporal dynamics of the mean OPI and its relationship with the mean EDI across 23 independent studies. Studies with higher EDI values are represented by larger and darker-colored points, reflecting periods of intense oviposition.

## 4. Discussion

The co-circulation and high number of reported cases of dengue, Zika, and chikungunya, along with the recent reemergence of Oropouche virus in Brazil, have contributed to increased morbidity and mortality, underscoring the need for mosquito surveillance strategies to monitor *Ae. aegypti*, *Ae. albopictus*, and other vectors in urban and periurban areas [50]. In response to the threat of urban arboviruses, the Brazilian Ministry of Health has promoted and monitored the development of new strategies for the entomological surveillance and control of *Ae. aegypti*. In its updated Guidelines for the Prevention and Control of Urban Arboviruses, the Ministry recommends the use of ovitraps as a core strategy for monitoring mosquito circulation [43,51]. Ovitraps are classical tools designed to attract and collect eggs from female mosquitoes seeking oviposition sites [31]. The primary aim of this strategy is to generate timely information on vector infestation to guide targeted control interventions. These guidelines also recommend ovitrap deployment for the initial territorial characterization, serving as a foundation for the introduction and assessment of innovative control technologies, including larvicide dissemination stations, *Wolbachia*-infected mosquitoes, and sterile insect techniques using irradiation [43]. To date, 457 Brazilian municipalities have incorporated ovitraps into routine *Aedes* surveillance, with 200 municipalities receiving targeted training to ensure correct implementation (personal communication with J.B.P. Lima, July 2025).

In this study, we conducted a systematic review of publications reporting Brazilian research that employed ovitraps to monitor *Aedes* populations, specifically *Ae. aegypti* and *Ae. Albopictus*, published from 2012 to 2022, with the aim of synthesizing surveillance data obtained through this oviposition method. Our findings showed that most articles included in this review were published in English rather than Portuguese, with notable variation across years. The highest number of publications occurred in 2020, with six in Portuguese and fourteen in total. This supports earlier observations by Di Bitetti et al. (2016), who reported that non-native English-speaking researchers often prioritize publishing in English-language journals, likely due to the expectation that such publications will achieve greater visibility and citation rates [52]. The data also indicated that bilingual publications (Portuguese and English) were rare, occurring only in 2017, 2019, and 2022, possibly reflecting limited uptake of dual-language dissemination strategies by Brazilian researchers. The observed temporal fluctuations in publication volume, with peaks in 2015, 2018, and 2020, may correspond to changes in research funding priorities or shifts in arboviral epidemiological trends in Brazil.

In our results, we found that most studies were concentrated in Brazil’s southeast and northeast regions, where scientific output on ovitrap use was higher than in other regions. In the southeast, 30 articles addressed topics such as *Aedes* vector infestation, trap comparisons, oviposition behavior, and *Wolbachia* monitoring. In the northeast, 31 articles examined areas including automated *Aedes* egg counting, vector control, and the effectiveness of biolarvicide-treated ovitraps. Beyond this geographic clustering, it is important to consider that the historical epidemiology of urban arboviruses shows that the northeast and southeast regions consistently report the highest number of probable dengue, Zika, and chikungunya cases and related fatalities, as documented by the Brazilian Ministry of Health [43,50]. However, potential bias must be considered regarding whether the higher ovitrap-related research output in these regions reflects actual arbovirus incidence or a concentration of research capacity.

Oviposition studies play a critical role in guiding evidence-based surveillance and vector control programs targeting disease vectors [53], particularly for monitoring arbovirus transmission in field-collected eggs of *Ae. aegypti* and *Ae. albopictus* [54]. In this review, we identified studies employing diverse ovitrap designs for mosquito monitoring, including traditional ovitraps (with water or hay infusion), ovitraps combined with larvicides, traps using plant extracts, water with cat food, and brewer’s yeast. Notably, studies reported increased efficacy when using hay infusions, reinforcing the importance of olfactory attractants in enhancing surveillance sensitivity [21,55].

Several studies included in this systematic review compared ovitraps with alternative mosquito surveillance methods. For instance, De Melo et al. (2012) assessed multiple *Ae. aegypti* surveillance tools and found that MosquiTRAP data showed a stronger temporal and spatial correlation with dengue fever cases compared to ovitraps and larval surveys, emphasizing the need for fine-scale risk assessment tools [56]. Similarly, Resende et al. (2013) compared MosquiTRAPs, ovitraps, and larval surveys, concluding that while each method had specific strengths and limitations, ovitraps and MosquiTRAPs demonstrated greater sensitivity than traditional larval surveys [57]. Codeço et al. (2015) evaluated larval surveys and four types of traps; ovitraps, MosquiTRAPs, Adultraps, and BG-Sentinels across five mid-sized Brazilian cities with diverse climates. Their results indicated that adult traps provided more sensitive and reliable data, and when used in combination, enhanced surveillance and control efforts synergistically [31]. Silva and Limongi (2018) compared four traps under field conditions and found that ovitraps had the highest positivity rates for detecting *Ae. aegypti*, although temperature and rainfall influenced trap effectiveness [58]. Monteiro et al. (2020) compared ovitraps with the Larval Index Rapid Assay (LIR*Aa*), concluding that ovitraps were useful but insufficient as standalone tools. Instead, combining ovitraps with LIR*Aa* improved overall surveillance effectiveness [59]. Finally, Jesus et al. (2022) found that ovitraps were as accurate as BG-Sentinels for estimating *Wolbachia* frequency during mass mosquito releases, while being significantly more cost-effective [60].

Previous studies, such as that by Braga et al. (2000), which compared larval surveys and ovitraps for monitoring *Ae. aegypti* populations, reported that ovitraps were more cost-effective, operationally viable, and highly effective for surveillance of this vector [29]. Gama et al. (2007) also compared MosquiTRAPs, ovitraps, and larval surveys in the state of Minas Gerais to identify the most effective monitoring tools during the dry season [61]. This study, along with others, including Braga et al. (2000), confirmed that ovitraps are particularly sensitive for detecting *Aedes* mosquitoes [29]. Moreover, Morato et al. (2005) concluded that ovitraps were easier to standardize than traditional larval indices in comparative evaluations [62]. Most of the selected studies focused predominantly on *Ae. aegypti*, highlighting a research gap concerning *Ae. albopictus*. This gap underscores the need for studies addressing both sympatric species, especially given their ecological overlap and mutual competence in transmitting dengue, chikungunya, and Zika viruses across endemic regions [1,3,63].

Despite their widespread use, the Breteau Index (number of containers with larvae per household) and the Building Infestation Index (number of positive properties relative to the total properties surveyed) have been questioned by some authors regarding their effectiveness. According to Rawlins (1998), in areas with low infestation levels, larval surveys alone are insufficient to detect the presence of vectors [64]. Similarly, Focks (2003) argued that larval indices are poor indicators of adult mosquito abundance [34]. Therefore, incorporating ovitraps into integrated vector management strategies are of high relevance. This method enables the calculation of entomological indicators such as the Egg Density Index (EDI) and OPI, which have demonstrated greater sensitivity in detecting early infestations [31,65]. Our findings indicate that high EDI values do not necessarily align with high OPI values, suggesting that these indicators capture distinct aspects of *Aedes* population dynamics. The potential to derive multiple indices from ovitrap data reinforces their utility as an essential tool in *Aedes* surveillance [66,67,68,69]. To ensure their effectiveness in Brazil, it is essential to adapt ovitrap implementation to regional contexts by considering both environmental characteristics and local climatic conditions [42,43,70]. Finally, based on the studies reviewed, ovitraps showed the highest sensitivity and strongest correlation with climatic variables, consistently reflecting adult mosquito population trends observed using adult traps [31]. Given the urgent need for sensitive, scalable surveillance tools in public and occupational health programs targeting *Ae. aegypti* and *Ae. albopictus*, ovitraps remain a reliable and cost-effective classical method.

## 5. Conclusions

The results of this systematic review reinforce the importance of ovitraps as a sensitive, scalable, and cost-effective tool for detecting the presence and estimating the relative abundance of both *Ae. aegypti* and *Ae. albopictus* in Brazil. Although all studies included in the review were conducted within the country, only 21.7% were published in Portuguese. This language imbalance may hinder effective communication with local communities, frontline health workers, and municipal decision-makers. Overall, the findings highlight the critical value of ovitrap-based entomological surveillance, offering actionable insights to guide the planning, implementation, and evaluation of vector control strategies in Brazilian urban and peri-urban settings.

## Data Availability

The data supporting this review are available within the article and its Supplementary Materials.

## Supplementary Materials

The following supporting information can be downloaded at https://www.mdpi.com/article/10.3390/tropicalmed10080212/s1, Table S1: Articles identified in the present review that reported on studies that targeted *Aedes aegypti* or *Aedes albopictus* or both study species (*Ae. aegypti* and *Ae. albopictus*) and had key words in Portuguese; Table S2: Articles identified in the present review that reported on studies that targeted *Aedes aegypti* or *Aedes albopictus* and both study species (*Ae. albopictus* and *Ae. aegypti*) and had key words in English. References [71,72,73,74,75,76,77,78,79,80,81,82,83,84,85,86,87,88,89,90,91,92,93,94,95,96,97,98,99,100,101,102,103,104,105,106,107,108,109,110,111,112,113,114,115,116,117,118,119,120,121,122,123,124,125,126,127,128,129,130,131,132,133,134,135,136,137,138,139,140,141,142,143,144,145,146,147,148,149,150,151,152,153,154] are cited in the supplementary materials.
