# Supplementary material for: Systematic Review of the Ovitrap Surveillance of Aedes Mosquitoes in Brazil (2012–2022)
Source: Trop Med Infect Dis. 2025 Jul 28;10(8):212. doi: 10.3390/tropicalmed10080212 (PMC12390002; doi:10.3390/tropicalmed10080212)
Supplement: Supplementary file 1 [file tropicalmed-10-00212-s001.zip › tropicalmed-3687599-supplementary.pdf]

Table S1. Articles identified in the present review that reported on studies that targeted *Aedes aegypti* or *Aedes albopictus* or both study species (*Ae. aegypti* and *Ae. albopictus*) and had key words in Portuguese.

Year	Article	Region[1]	Language	Objective	Species	Principal findings	Reference
2012	Oliveira & Lima	Southeast /MG	Portuguese	Establish and train the "Mirim Agents Environment Brigade", a group of elementary school students and teachers.	*Aedes aegypti* and *Ae. albopictus*	The brigade was formed, qualified, consolidated, and made responsible for the inclusion of local residents, students, and school teachers, and partnerships and strategies of the KAP (Knowledge, Attitudes and Procedures about the disease) for the control of *Aedes*, through the installation and weekly monitoring of ovitraps.	[71]
2012	Silva et al.	Northeast/PE	Portuguese	Develop an optical platform system for automatic egg counting	*Aedes aegypti*	Two prototypes of the proposed system were installed and implemented in two municipalities in Pernambuco state.	[72]
2013	Oliveira & Musis	Central West/MT	Portuguese	Analyze the influence of abiotic factors on the fluctuations in the *Ae. aegypti* and *Ae. albopictus* populations at a local school between January and August 2012, considering the microclimatic variations at the study site.	*Aedes aegypti* and *Ae. albopictus*	Eggs were detected in all samples and at all sampling points. The entomological indicators show a high level of infestation in the study area.	[73]
2013	Serpa et al.	Southeast/SP	English	Study the spatial distribution of *Ae. aegypti* and *Ae. albopictus* in the municipality.	*Aedes aegypti* and *Ae. albopictus*	Habitat segregation was recorded in *Ae. aegypti* and *Ae. albopictus*, which may reflect the influence of local environmental features and interactions between the species.	[74]
2016	Bellinato et al.	North, Southeast, Central west/RR, PA, RN, ES, GO	English	Test the resistance of laboratory-raised adult *Ae. aegypti* to the insecticides Temephos, Deltamethrin, and Diflubenzuron.	*Aedes aegypti*	In some municipalities, the application of insecticides was related to a high incidence of dengue.	[75]

2016	Costa et al.	Northeast/BA	Portuguese	Describe the seasonal distribution of *Ae. aegypti* and *Culex quinquefasciatus* and the incidence of dengue in the municipality.	*Aedes aegypti*	The occurrence patterns of the two species may be related to the interaction of a set of factors (competition, demographic parameters, and climatic conditions).	[76]
2016	Depoli et al.	South/PR	Portuguese	Use of different attractants for *Aedes* surveillance and control.	*Aedes aegypti* and *Ae. albopictus*	The study verified the efficiency of the ovitraps for the monitoring of *Ae. aegypti* and *Ae. albopictus* in an urban area.	[77]
2017	Barbosa et al.	Northeast/RN	English and portuguese	Monitor the *Ae. aegypti* population in the municipality.	*Aedes aegypti*	The ovitraps were presented as viable traps for monitoring *Ae. aegypti.*	[66]
2018	Alves et al.	Northeast /CE	English	Collection of *Ae. aegypti* eggs to verify the impact of plant extracts on these eggs.	*Aedes aegypti*	Ovitraps that contained dirty water (sewage) and guava extract were the most attractive for oviposition.	[78]
2018	Silva & Limongi	Southeast /MG	Portuguese	Compare different trapping methods for the collection of *Ae. aegypti*.	*Aedes aegypti*	In 500 surveys, 20 adult *Ae. aegypti* (30% females) collected in MosquiTRAP traps, 5754 eggs in the ovitraps (panel/filter paper), and 47 larvae in mosquito nets. Only *Ae. aegypti* recorded. Ovitraps provided the best indices for the surveillance of this vector, in particular in the dry season. The use of filter paper as an oviposition substrate was also recommended.	[58]
2018	Zequi et al.	South/PR	Portuguese	Estimate the variation in the *Ae. aegypti* and *Ae. albopictus* populations in relation to urbanization and assess the overlap and competition between these species.	*Aedes aegypti* and *Ae. albopictus*	Both vectors were recorded in all the areas in which the traps were deployed, with the probable overlap of the species in the area.	[35]
2019	Barbosa et al.	Southeast/SP	English and portuguese	Evaluate whether sites with more potential *Ae. aegypti* breeding grounds influence the active dispersal of the vector to the surrounding area.	*Aedes aegypti*	No confirmation of any differential role of the strategic points in the dispersal of the vector to neighboring properties.	[79]

2019	Custodio et al.	Central west/MS	English	Analysis of the behavior of *Ae. aegypti* and *Ae. albopictus* in the municipality	*Aedes aegypti* and *Ae. albopictus*	The ovitraps recorded high frequencies of *Ae. aegypti* and the presence of *Ae. albopictus* in urban areas.	[80]
2019	Fonseca et al.	Northeast/BA	Portuguese	Obtain larvae to test the larvicides Pyriproxyfen, Novaluron, and Spinosad	*Aedes aegypti*	The ovitrap-type traps were efficient for the collection of *Ae. aegypti* eggs. The experiment confirmed that all three products controlled the mosquito effectively.	[81]
2019	Heinisch et al.	Southeast/SP	Portuguese	Monitoring of *Ae. aegypti* and *Ae. albopictus* during the spring and autumn.	*Aedes aegypti* and *Ae. albopictus*	The ovitrap data on *Ae. aegypti* and *Ae. albopictus* supported the conclusion that the seasonal variation in abundance is linked directly to ambient temperatures, which affect oviposition directly.	[67]
2019	Gonçalves e Sá et al.	Northeast/PE	Portuguese	Evaluate the local infestation of *Ae. aegypti* using ovitraps in the Santa Margarida neighborhood.	*Aedes aegypti*	Monitoring by ovitraps and LIR*Aa*, over one year, found that high precipitation and relative humidity are the factors that contribute most to the proliferation of *Ae. aegypti* in the study area, with the number of eggs increasing between March and June 2018, after the longest rains. The incidence of eggs (traps) and larvae (LIR*Aa*) was highest in May.	[82]
2020	Ferreira et al.	Northeast/MA	Portuguese	Test the larvicidal potential of different extracts of *Aniba rosaeodora* Ducke.	*Aedes aegypti*	The ovitrap-type traps were efficient for the collection of *Ae. aegypti* eggs. The experiment confirmed that the Linalool extract had the most significant larvicidal potential.	[83]
2020	Mafra et al.	Northeast/MA	Portuguese	Test the larvicidal effect of different extracts of *Ocimum basilicum* Linn.	*Aedes aegypti*	The ovitrap-type traps were efficient for the collection of *Ae. aegypti* eggs. The experiments confirmed that the extract of *Ocimum basilicum* Linn had considerable larvicidal potential.	[68]
2020	Martins et al.	Northeast/MA	Portuguese	Test the larvicidal effect of different extracts of *Pimenta dioica* Lindl.	*Aedes aegypti*	The ovitrap-type traps were efficient for the collection of *Ae. aegypti* eggs. The experiments confirmed the extract of *Pimenta dioica* Lindl. had considerable larvicidal potential.	[69]

2020	Monteiro et al.	Northeast/PI	Portuguese	Comparative analysis of the effectiveness of ovitraps and the Larval Index Rapid Assay (LIR*Aa*) method for the assessment of the presence of *Ae. aegypti.*	*Aedes aegypti*	Monitoring the mosquito should not depend solely on ovitraps, but should be combined with the LIR*Aa* method for best results.	[59]
2020	Moraes et al.	Southeast/MG	Portuguese	Evaluate the proliferation of *Ae. aegypti* to indicate the presence of the mosquito in the location and generate the risk map.	*Aedes aegypti*	The ovitraps were useful for the monitoring of the proliferation of the mosquito.	[84]
2020	Oliveira et al.	Central West/MT	Portuguese	Monitor *Ae. aegypti* and *Ae. albopictus*	*Aedes aegypti* and *Ae. albopictus*	The black ovitrap was the most efficient for the monitoring of both species, but it was most effective for *Ae. albopictus* at both ground level and 1.5 m, it was only effective for *Ae. aegypti* at 1.5 m.	[85]
2021	Andrade et al.	Northeast/BA	Portuguese	Collection of *Ae. aegypti* eggs to verify the impact of *Phyllanthus acuminatus* Vahl extract on the larva.	*Aedes aegypti*	The extract of *Phyllanthus acuminatus* Vahl. presented a clear larvicidal effect on *Ae. aegypti.*	[86]
2021	Júnior et al.	Northeast/PE	Portuguese	Spatial analysis of dengue cases in the municipality.	*Aedes aegypti*	The data from the ovitraps were fundamental to the spatial analysis. There was a significant correlation between two meteorological variables (ambient temperature and relative humidity) and the number of eggs, whereas precipitation did not correlate systematically, which indicates that, in the semi-arid region, the prevalence of the insects is most closely related to the way water is stored for human activities.	[87]

[1] State abbreviations: AM – Amazonas, AP – Amapá, BA – Bahia, CE – Ceará, DF – Distrito Federal, ES – Espírito Santo, GO - Goiás, MA – Maranhão, MG – Minas Gerais, MS – Mato Grosso do Sul, MT – Mato Grosso, PA – Pará, PE – Pernambuco, PI – Piauí, PR – Paraná, RJ – Rio de Janeiro, RN – Rio Grande do Norte, RR – Roraima, SP – São Paulo, SE – Sergipe, TO – Tocantins

Table S2. Articles identified in the present review that reported on studies that targeted *Aedes aegypti* or *Aedes albopictus* and both study species (*Ae. albopictus* and *Ae. aegypti*) and had key words in English.

Year	Article	Region[1]	Language	Objective	Species	Principal findings	Reference
2012	Campos et al.	Southeast/ SP	English	Investigate *Ae. aegypti* population dynamics under marked seasonal climatic variation and determine the associated seasonal variation in the genetic diversity of the vector.	*Aedes aegypti*	This study supports the implementation of vector control measures targeting immature stages of *Ae. aegypti* during the dry season. Although eliminating breeding sites is virtually impossible during the rainy season, the concentration of oviposition at fewer breeding sites during the dry season may increase the overall feasibility of this control strategy.	[88]
2012	De Melo et al.	Southeast /MG	English	Evaluate how different methods of *Ae. aegypti* surveillance correlates with the occurrence of dengue fever in a pre-selected area in 2007–2008.	*Aedes aegypti*	The MosquiTRAP data correlated more closely with the distribution of dengue fever, both temporally and spatially, in comparison with larval surveys and ovitraps. To improve vector control, surveillance tools capable of the fine scale assessment of the risk of transmission of dengue are necessary.	[56]
2013	Gambarra et al.	Northeast/ PB	English	Determine resistance to temephos and evaluate esterase activity in resistant *Ae. aegypti* populations, to help state health officials develop better strategies for the monitoring and control of the populations of this vector.	*Aedes aegypti*	The study confirmed the ample distribution of *Ae. aegypti* and its high infestation indices (OTIs) in six municipalities, as well as resistance to temephos (Resistance Rate [RR] > 20).	[89]
2013	Oliveira & Musis	Central West/MT	Portuguese	Analyze the influence of abiotic factors on the fluctuations in the *Ae. aegypti* and *Ae. albopictus* populations at a local school between January and August 2012, considering the	*Aedes aegypti* and *Ae. albopictus*	Eggs were detected in all samples and at all sampling points. The maximum OPI (88.9%), EDI (11.1%), and VDI (192.3 eggs/arm) all indicated a high level of infestation in the study area.	[73]

				microclimatic variations at the study site.			
2013	Padilla- Torres et al.	North/AM	English	Quantify the effects of routine control interventions and selected environmental variables on the temporal variation in dwelling infestation rates, the principal indicator used in vector control programs.	*Aedes aegypti* and *Ae. albopictus*	The study identified two strategies for improving dengue vector surveillance: (i) ovitraps baited with a hay infusion are preferable to rapid larval surveys due to their greater sensitivity and potential for measuring the infestation of dwellings by foraging gravid females, (ii) the repeated-sampling approach used in study improves considerably the estimates of infestation rates by taking imperfect detection into account explicitly.	[90]
2013	Peres et al.	Southeast/ RJ	English	Evaluate the possible correlation between buildings with higher Premise Condition Index (PCI) scores and the number of *Ae. aegypti* eggs and adults collected.	*Aedes aegypti*	More *Ae. aegypti* eggs were collected in the ovitraps that were installed in houses with a higher PCI score.	[91]
2013	Piovezan et al.	Southeast/ SP	English	Evaluate the spatial distribution and diversity of immature mosquitoes in a mixture of Cerrado savanna and Atlantic Forest ecosystems, using ovitraps and larvitraps.	*Aedes aegypti* and *Ae. albopictus*	As the eggs of *Ae. aegypti* are more resilient to environmental change and this species prevails in more urbanized environments, the higher concentration of larvae recorded in the northeast of the study neighborhood raised questions on the determinants.	[92]
2013	Regis et al.	Northeast/ PE	English	Evaluate and improve the applicability of an environmentally-friendly vector monitoring and control system that aims to contribute to a reduction in the transmission of the dengue virus.	*Aedes aegypti*	The authors concluded that the mass elimination of *Aedes* eggs should be considered because it may prevent population booms resulting from the sudden hatching of large amounts of eggs that remain dormant during the dry season. Ovitraps are an ideal monitoring tool because they are simple to handle and favor social engagement.	[93]

2013	Resende et al.	Southeast /MG	English	Compare the MosquiTRAP (an adult sticky trap) with ovitraps and larval surveillance.	*Aedes aegypti*	Each of the methods tested presented specific advantages and disadvantages in terms of their sensitivity and operational use. In comparison with larval surveys, the ovitraps and MosquiTRAPs were more sensitive.	[57]
2014	Lana et al.	Southeast/ RJ	English	Assess the validity of ubiquitous biological assumptions in the dengue modeling literature and discuss the impact of the observed patterns on the dynamics of dengue fever in Rio de Janeiro.	*Aedes aegypti*	More than 90% of traps detected oviposition, with the suburban slum having the lowest mosquito population density and the strongest seasonality. This slum is densely populated, with an accumulation of garbage in its streets.	[94]
2014	Leal-Santos et al.	Central West/MT	English	Assess the level of *Ae. aegypti* infestation and the influence of abiotic factors (temperature, humidity, and rainfall) in the internal and external areas of the university hospital.	*Aedes aegypti*	Hospitals may be important locations for the dissemination of dengue and should thus be considered to be strategic areas in dengue control programs.	[95]
2014	Linns et al.	All regions of Brazil/SE, GO,PA,RR ,RN,MS,E S,RJ,AL,R S	English	Investigate the presence of genetic mutations ('knockdown resistance' or kdr mutations) in *Ae. aegypti* that affect the susceptibility of this species to pyrethroids.	*Aedes aegypti*	As *Ae. aegypti* is essentially an urban mosquito, it is exposed to strong pyrethroid selection, which means that many populations are becoming resistant to this class of insecticides. Knowledge of the diversity of the sodium channel in natural *Ae. aegypti* populations together with the role of each allele in pyrethroid resistance and their effects on fitness will be crucial to ensure the effectiveness of pyrethroid as a viable control agent.	[96]
2014	Monteiro et al.	North /AP	Portuguese	Monitor the distribution of *Ae. aegypti* oviposition and its temporal dynamics and correlate the findings with the data on environmental variables.	*Aedes aegypti*	Macapá is infested by *Ae. aegypti*, whose high densities are influenced strongly by environmental variables, with the highest entomological indices occurring during the rainy season.	[97]

2014	Pessanha et al.	Southeast /MG	English	Evaluate how effective the ovitrap is as a surveillance tool for the assessment of the risk of human dengue infection	*Aedes aegypti*	Surveillance using the ovitrap, a simple and easily adaptable device, provides a powerful tool for the prediction of dengue outbreaks, with a two-month early warning time.	[98]
2014	Regis et al.	Northeast/ PE	English	Determine the temporal and spatial distribution of *Ae. aegypti* in 15 of the island's villages.	*Aedes aegypti*	A population of *Ae. aegypti* that has been established throughout the inhabited areas of the island, with varying population densities, which reflect seasonal and spatial patterns.	[99]
2014	Santos et al.	Northeast/ PE	English	Evaluate the efficacy of the use of *Panicum maximum* (WSMoL) in traps to capture adult female *Ae. aegypti* or eggs under semi-field conditions.	*Aedes aegypti*	The WSMoL was effective as a stimulant of oviposition, increasing the capture of eggs in the ovitraps under semi-field conditions. But it did not increase the number of females captured in the adult traps.	[100]
2015	Abreu et al.	Southeast /MG	English	Determine the number of sites used by each female to lay eggs and how this varies according to the availability of oviposition sites and local conditions.	*Aedes aegypti*	The disproportional distribution of eggs demonstrated the existence of preferred breeding sites. The preferred breeding site was that which received the highest percentage of eggs. These sites were observed under both semi-field and laboratory conditions.	[101]
2015	Arduino et al.	Southeast/ SP	English	Comparative evaluation of the biological cycles of *Ae. aegypti* populations under varying conditions of salinity.	*Aedes aegypti*	Both *Ae. aegypti* study populations (coastal and plateau) were able to oviposit in brackish water at salt concentrations of up to 17‰ and to develop at concentrations of up to 14‰.	[14]
2015	Brasil et al.	Central West/DF	English	Introduce the complete Digital Image Processing (DIP) algorithm for the counting of the eggs deposited in the ovitrap, integrated with a web-based SemiAutomatic Counting System (SCSA), and automatic georeferencing of the data (GIS).	*Aedes aegypti*	The DIP algorithm confirms the potential for an efficient automatic egg count. The automated egg counts were highly similar to the manual counts, which validates the Java program application.	[102]

2015	Carvalho et al.	Northeast/ BA	English	Describe a field evaluation of one prominent transgenic-vector strategy, the use of male mosquitoes carrying a lethal or autocidal transgene in a sterile- male-release system.	*Aedes aegypti*	The long-term goal for vector control should be maintenance below the transmission threshold, despite the low herd immunity. The data indicate that the release of OX513A males can achieve this goal.	[103]
2015	Cecílio et al.	Southeast /MG	English	Assess the circulation of *Ae. aegypti* and *Ae. albopictus*, and detect dengue virus transovarial transmission.	*Aedes aegypti* and *Ae. albopictus*	Using real time PCR, DENV was detected in four of the 54 pools of *Ae. aegypti* larvae hatched in the laboratory. Despite the low rate of vertical transmission the positivity of the pools was high (7.41%) and the results were passed on to the local health surveillance departments to ensure better intervention at these locations.	[104]
2015	Chapadense et al.	Central west/GO	English	Determine the tolerance of deltamethrin in three *Ae. aegypti* populations and the frequency of the 1016 and 1534 polymorphisms of the AaNa(V) gene in these populations.	*Aedes aegypti*	The results indicate that pyrethroids are no longer effective for suppressing *Ae. aegypti* in Goiás. These findings highlight the urgent need for the implementation of alternative and/or complementary methods of vector control.	[105]
2015	Codeço et al	North, Southeast, Central west, Northeast/ PA, RN, RJ, MS	English	Compare the standard larval survey with four types of trap: ovitrap, MosquiTRAP, Adultrap, and BioGent-Sentinel.	*Aedes aegypti*	Traps rarely produced null indices, pointing to their greater sensitivity in detecting the presence of *Ae. aegypti* in comparison to the larval survey. Traps are useful for monitoring adult infestation levels and the impact of control strategies. Used together, they optimize surveillance, prevention, and control synergistically.	[31]
2015	Cruz et al.	Central West/MT	English	Estimate the rates of natural transovarial infection by *Flavivirus* in an area with a high incidence of acute febrile illness suspected to be caused by dengue.	*Aedes aegypti*	The study detected natural transovarial infection of DENV-4 in *Ae. aegypti* in Cuiabá, with a relatively high MIR index, which favors the birth of mosquitoes infected with DENV at the beginning of an epidemic.	[106]

2015	Dias et al.	Northeast/ MA	English	Test the larvicidal activity of essential oils extracted from five common plant species found in the Brazilian Legal Amazon region, and determine the chemical composition of the oils, to establish parameters of quality control.	*Aedes aegypti*	The *Ae. aegypti* larvae were sensitive to four of the five essential oils tested.	[107]
2015	Santana et al.	North/RO	English	Evaluation of the larvicidal potential of the essential oils of three *Piper* species commonly found in the Brazilian Amazon region for the control of *Ae. aegypti*	*Aedes aegypti*	The principal components identified in *Piper arboreum* were germacrene D (31.83%) and bicyclogermacrene (21.40%), in *Piper marginatum*, they were (E)-methyl isoeugenol (27.08%), (E)-anethole (23.98%), and (Z) - methyl isoeugenol (12.01%), while in *Piper aduncum*, they were (E) - Isocroweacin (29.52%), apiol (28.62%), and elemicin (7.82%). The essential oils from the study species had lethal concentrations (LC50) at 34–55 ppm, with LC90 = 85 ppm in P. marginatum, and over 100 ppm in the other species. The essential oil of P. marginatum was the most effective larvicide against Ae. aegypti.	[108]
2015	Schultes et al.	Southeast /MG	English	Assess the relationship between the spatial patterns of dengue incidence and the *Ae. aegypti* ovitrap positivity index (OPI) over time.	*Aedes aegypti*	The transmission of dengue is influenced by a complex combination of factors, with the spatial distribution of cases changing over 12 years. The shifting spatial association between *Ae. aegypti* eggs and the incidence of dengue indicates that the data on the vector alone may not account for the spatial patterns in the incidence of dengue.	[109]

2015	Soares et al.	Southeast /MG	English	Evaluate the egg-laying behavior of *Ae. aegypti* in ovitraps and its relationship with climatic factors in a semi-arid region.	*Aedes aegypti*	Ovitraps made from recyclable material may be a useful and environmentally-friendly tool for studying the behavior of Ae. aegypti. The deposition of eggs occurred homogeneously in the two study neighborhoods, indicating behavioral homogeneity in the local population.<br>Climatic factors, especially rainfall and relative humidity influenced directly the entomological indices and mosquito infestation.	[110]
2015	Taranto et al.	Southeast /MG	English	Analysis of the possible correlation between the incidence of *Aedes* spp. and climatic, socio-economic, and demographic parameters, as well as confirmed dengue cases.	*Aedes aegypti* and *Ae. albopictus*	*Aedes albopictus* is common in the study area, with seasonal and spatial variation being found in the distribution of both *Ae. aegypti* and *Ae. albopictus*. In particular, the local prevalence of *Ae. albopictus* exceeded the expected levels, which is consistent with the findings of previous studies, worldwide.	[111]
2015	Wermelinger et al.	Southeast/ RJ	English	Describe *Ae. aegypti* oviposition patterns in a large urban area during hot weather and identify the possible determinants of these patterns.	*Aedes aegypti*	Eggs on the surface of water should be considered during entomological surveillance, especially during dry periods. These findings also indicate that *Ae. aegypti* females may often oviposit some of their eggs on the water surface in at least some natural breeding sites.	[112]
2016	Aguirre-Obando et al.	South/PR	English	Evaluate whether the resistance of *Ae. aegypti* populations to PY and OP Temephos is influenced by the intensification of vector control.	*Aedes aegypti*	The rapid increase of the $1016Ile^{kdr}$ allele in natural *Ae. aegypti* populations emphasizes the need for the development of alternative strategies such as insecticide rotations and mixtures to delay the evolution of resistance. The results of the study reinforce the need for preventive, rather than isolated emergency strategies of vector control. Without long-term data, resistance to insecticides may be maintained.	[113]

2016	Chediak et al.	All regions of Brazil/All States	English	Understand the spatial and temporal dispersal of temephos resistance in Brazil over the preceding 12 years.	*Aedes aegypti*	While the frequency of temephos resistance appeared to be limited in the principal problem areas in 2004–2005, resistance continued to spread across Brazil. By 2010– 2011 temephos presented satisfactory results against YF mosquito larvae in only Rondônia (North), São Paulo (Southeast), Paraná and Santa Catarina (South) states. New focal areas of temephos resistance were detected in 2010–2011 radiating from near Rio Branco (southern Acre in North Brazil) and Brasilia (central Brazil), indicating the country-wide spread of resistance. New focal areas of temephos resistance were detected in 2010–2011 radiating from near Rio Branco (southern Acre in North Brazil) and Brasilia (central Brazil), indicating the	[114]
2017	Barbosa et al.	Northeast/ RN	English and portuguese	Monitor the *Ae. aegypti* population in the municipality.	*Aedes aegypti*	The ovitraps were presented as viable traps for monitoring *Ae. aegypti.*	[66]
2017	Costa et al.	North/AM	English	Identify the circulating DENV serotypes in the larvae collected from four municipalities to provide a proposal for the use of the frequency of these serotypes as a tool for xenomonitoring.	*Aedes aegypti*	The study confirmed the potential of ovitraps as a monitoring tool for vectorial surveillance and viral monitoring. Also, can amplify disease monitoring systems in risk areas, to ensure the early detection of the virus, contributing to more effective control measures.	[115]
2017	Dias et al.	Northeast, Southeast, Central west and North/AL, BA, ES, GO, PA, SP	English	Assess and compare the toxicity of spinosad and temephos insecticides in Brazilian *Ae. aegypti* populations with putative different genetic backgrounds, under laboratory and field (simulated) conditions.	*Aedes aegypti*	No cross-resistance of spinosad with temephos was recorded, regardless of the resistance level to the organophosphate.	[116]

2017	Fontoura et al.	Northeast, Southeast and Central west/CE, SE, MG, MT	English	Quantify the effect of novaluron in Rockefeller, a reference *Ae. aegypti* strain susceptible to insecticides.	*Aedes aegypti*	No significant differences in novaluron persistence were found among localities, or between them and the Rockefeller mosquitoes. In all cases, larval mortality was significantly higher after the 5th day of contact with novaluron.	[117]
2017	Hendy et al.	North/AM	English	Verify the need for diverse sampling approaches for the understanding of the spatiotemporal variation in diurnal anthropophilic mosquitoes, using BG-Sentinel traps.	*Aedes aegypti*	The study identified several potential routes of transmission for mosquito-borne viruses between the ground and forest canopy.	[118]
2017	Prado et al.	Southeast/ES	English	Evaluate the frequency of oviposition of *Ae. aegypti* at the different sites, under different shading conditions and pedestrian traffic in the vicinity of the traps, to provide insights into the behavior of this important arbovirus vector.	*Aedes aegypti*	Pedestrian traffic and shading only influenced positive traps when their indices were lower than 0.1. This points to a relationship of interdependence, that is, positivity is affected only when both factors were intense and frequent.	[119]
2018	Albuquerque	North/AM	English	Analyze the relationship between the occurrence of dengue and ovitrap positivity and egg density recorded in 2016.	*Aedes aegypti*	The distribution of the cases of dengue is related to ovitrap egg positivity, and less clearly, to egg density. The regular distribution of ovitraps in the urban zone of the study municipalities permitted the identification of the potential local risk of the occurrence of dengue.	[120]
2018	Ayllon et al.	Southeast/RJ	English	Determine the degree of adaptation of the *Ae. albopictus* to a domestic lifestyle in urban slums.	*Aedes albopictus*	*Aedes albopictus*, a competent vector for important arboviruses, including dengue (DENV), chikungunya (CHIKV), Zika (ZIKV), and yellow fever (YFV), may spread into neglected and densely urbanized areas, in close proximity to areas of natural vegetation. These findings emphasize the global importance of the inclusion of the	[121]

						monitoring of *Ae. albopictus* in surveillance and control programs.	
2018	Costa et al.	North/AM	English	Determine the natural vertical transmission of arboviruses during the emergence of Zika (ZIKV) in 2015–2016.	*Aedes aegypti*	The first detection of the natural vertical transmission of ZIKV in *Ae. aegypti*, which may contribute to the maintenance of ZIKV in the wild during epidemics.	[54]
2018	Garcia et al.	North, Northeast, Southeast and Central west/PR,RN,RJ,MS	English	Evaluate the dynamics of the resistance of *Ae. aegypti* populations in four Brazilian regions over the course of one year.	*Aedes aegypti*	Seasonal increase in the domestic use of pyrethroidinsecticides, occurring mainly during outbreaks, accompanied by a seasonal elevation of resistance levels. The results highlighted the limitations of chemical substances as the principal strategy for the control of *Ae. aegypti* larvae and adults.	[122]
2018	Garziera et al.	Northeast/MA,BA	English	Assess the related changes in the distribution of the infestation and abundance of *Ae. aegypti* populations 6 and 18 months after the release of transgenic mosquitoes.	*Aedes aegypti*	Genetic controls associated with traditional methods reduced the *Ae. aegypti* population effectively. The study also showed that populations can be reestablished when only a conventional control method is used.	[123]
2018	La Corte et al.	Northeast/SE	English	Provide a baseline for the evaluation of the evolution of susceptibility to organophosphates in the absence of selection pressures.	*Aedes aegypti*	The variation in resistance levels observed in local *Ae. aegypti* populations highlights the need for an effective program of resistance surveillance, and also indicate that the potential of small urban centers to generate and spread insecticide resistance may have been underestimated.	[124]

2018	Dos-Santos et al.	North, Southeast, Central West/AM, RJ, GO	English	Evaluate the ability of *Ae. albopictus* to colonize and disperse in forest environments, ranging from edge/modified to more preserved forest in Brazilian biomes: Amazon, Cerrado, and Atlantic Forest. Analyze blood meals to determine *Ae. albopictus* feeding patterns and potential interactions with wild vertebrates.	*Aedes albopictus*	The study highlights the potential role of *Ae. albopictus* as a bridge vector for zoonotic diseases in the human-animal interface at the margins of Brazilian forests. The potential participation of the mosquito in the spill-over of dozens of zoonotic arboviruses found in Brazilian forests from sylvatic or ubiquitous hosts directly to humans or domestic hosts, and in other conserves, demands further investigation.	[125]
2018	Sacramento et al.	Northeast /CE	English	Estimate the associated factors of dengue seroprevalence among native Indians of the Tremembé ethnic group of Tapera village and their knowledge about the aspects related to the presence of mosquitoes of the genus *Aedes*	*Aedes aegypti*	The ovitrap positivity index increased from March through August, indicating an increase in the spatial distribution of the eggs. This is important because it identifies the period when there is a greater likelihood of contact between people and insects, which favors transmission.	[126]
2018	Silva et al.	North/AM	English	Analyze the effectiveness of different control agents associated with ovitraps for *Ae. aegypti* and *Ae. albopictus* under laboratory and field conditions.	*Aedes aegypti* and *Ae. albopictus*	Ovitraps associated with Vectobac®WG – *Bacillus thuringiensis israelensis* played an important role in the oviposition patterns of *Ae. aegypti* and *Ae. albopictus.*	[127]
2018	Zequi et al.	South/PR	Portuguese	Estimate the variation in the *Ae. aegypti* and *Ae. albopictus* populations in relation to urbanization and assess the overlap and competition between these species.	*Aedes aegypti* and *Ae. albopictus*	Both vectors were recorded in all the areas in which the traps were deployed, with the probable overlap of the species in the area.	[35]
2019	Barbosa et al.	Southeast/ SP	English and portuguese	Evaluate whether sites with more potential *Ae. aegypti* breeding grounds influence the active dispersal of the	*Aedes aegypti*	No confirmation of any differential role of the strategic points in the dispersal of the vector to neighboring properties.	[79]

				vector to the surrounding area.			
2019	Noleto et al.	Northeast/ MA	English	Demonstrate the potential of ovitraps for monitoring the spatial and seasonal infestation of *Aedes* mosquitoes in an area endemic for arboviruses.	*Aedes aegypti* and *Ae. albopictus*	The ovitraps were able to describe, reliably and at low cost, the infestation patterns of *Aedes* spp. in the study area. Rainfall was the principal abiotic factor affecting the breeding rate of these insects, even in the climatic context of the Cerrado savanna.	[128]
2019	Piovezan et al.	Southeast/ SP	English	Evaluate the use of ovitraps as an additional method for entomological surveillance in the study area, and determine the influence of the distribution of sites used to store recyclable materials on the occurrence of *Ae. aegypti* larval habitats.	*Aedes aegypti*	Ovitrap monitoring systems are sensitive and agile, and their routine inclusion in dengue control actions requires few resources. Further studies should determine parameters and guidelines for the implementation of measures based on the number of eggs found in a trap or in a given area.	[129]
2019	Sá et al.	Northeast/ PE	Portuguese	Evaluate the local infestation of *Aedes aegypti* using ovitraps and a simplified sampling method, the *Ae. aegypti* infestation index.	*Aedes aegypti*	Monitoring by ovitraps and LIR*Aa*, over one year, found that high precipitation and relative humidity are the factors that contribute most to the proliferation of *Ae. aegypti* in the study area, with the number of eggs increasing between March and June 2018, after the longest rains. The incidence of eggs (traps) and larvae (LIR*Aa*) was highest in May.	[82]
2019	Sá et al.	North/TO	English	Expand the known resistance profile of the *Ae. aegypti* populations from Tocantins state, identify the mechanisms underlying their resistance, and generate data that can help control programs develop more efficient strategies and operational procedures to combat the vector.	*Aedes aegypti*	The response to reports of dengue is vector control programs, most of which use insecticides. The fear of the disease also results in an increase in the use of domestic insecticides. Higher levels of insecticide resistance were thus expected in areas with a longer history of mosquito infestation and the presence of the arbovirus.	[130]

2019	Saraiva et al.	North /AP	English	Report the occurrence of *Ae. albopictus* in the state of Amapá.	*Aedes albopictus*	The record of *Ae. albopictus* reinforces the need for the surveillance of this mosquito vector in the Amazon region, where 34 arbovirus serotypes of medical importance are found.	[131]
2019	Soares et al.	Northeast/ PE	English	Evaluate the spatial distribution of *Ae. aegypti* in areas that are vulnerable to the transmission of arboviruses, and assess the influence of climatic conditions on the infestation of the mosquitoes.	*Aedes aegypti*	Several areas of risk for the transmission of arboviruses were identified, and climatic conditions influenced the infestation of *Ae. aegypti* during the rainy months. Ovitraps associated with entomological indices, climatic factors and georeferencing tools were identified as tools for the assessment of the local dispersal of the vector, and the development of preventive measures.	[132]
2020	Alencar et al.	Southeast/ RJ	English	Determine the occurrence of *Ae. aegypti* in a protected area.	*Aedes aegypti*	Wild populations of *Ae. aegypti* were identified, indicating a potential environment for the viral transmission of numerous arboviruses.	[133]
2020	Barbosa et al.	Northeast/ PE	English	Investigate the infestation patterns of *Ae. aegypti* and *Ae. albopictus* in the study area in 2013, in comparison with a previous assessment of this area, in 2005.	*Aedes aegypti* and *Ae. albopictus*	The mean number of eggs at the trapping stations where *Ae. aegypti* was predominant was much higher than those at which *Ae. albopictus* was the dominant species.	[134]
2020	Carvalho et al.	Central West/DF	English	Monitor the resistance of laboratory- raised adult *Ae. aegypti* to the insecticide Pyriproxyfen	*Aedes aegypti*	The Brazilian Ministry of Health uses LIR*Aa* to direct actions for the control of *Ae. aegypti*, breeding sites treated with PPF, in which the larvae remain alive, may lead to incorrect estimates of mosquito infestation levels, and the possibility of overlapping the treatment of *Ae. aegypti* larval.	[135]
2020	Jesus et al.	Southeast/ RJ	English	Comparative evaluation of the effectiveness of BG-Sentinel and ovitraps for the monitoring of the frequency of *Wolbachia*	*Aedes aegypti*	The ovitrap data estimated the frequency of *Wolbachia* as accurately as BG-Sentinels, which are 220 times more expensive. A random sample of 35% of the eggs collected in the ovitraps produced a similar invasion curve to	[60]

				during mass releases of mosquitoes.		that produced by screening all the adults collected in the BG-Sentinels.	
2020	Leandro et al.	South/PR	English	Evaluate the resistance of the local *Ae. aegypti* population to the insecticide malathion, under field and laboratory conditions, by comparing scenarios with and without the application of the insecticide.	*Aedes aegypti*	The relationship between real time entomological surveillance, using adult and egg traps in the field (Adultrap® and ovitraps), and the precision of laboratory measurements, is important for decision- making in the protection of human populations from arboviruses. The most important limitation is the use of the insecticide without any real-time knowledge of the actual susceptibility of the target *Ae. aegypti* population.	[136]
2020	MacCormack-Gelles et al.	Northeast /CE	English	Analysis of data on dengue cases in 2012–2015 (including both epidemic and interepidemic years) at the finest possible spatial scale.	*Aedes aegypti*	House indices measured by the LIR*Aa* did not predict reliably the scale or location of dengue transmission. Considering financial constraints, it is recommended that regional health secretariats examine critically the limitations of rapid larval surveys for the control of *Ae. aegypti*.	[137]
2020	Maia et al.	Southeast/ RJ	English	Assess and compare different sampling methods of a mosquito community in a fragment of the Atlantic Forest.	*Aedes albopictus*	The study concluded that the sampling methods for the collection of immature mosquitoes provide important indicators that should be included in efforts to monitor biological vectors. Tire traps had the highest colonization rates, followed by ovitraps.	[138]
2020	Moura et al.	Northeast/ RN	English	Describe the infestation of urban areas by evaluating the temporal and spatial distribution of *Ae. aegypti* and *Ae. albopictus* oviposition between 2016 and 2018, and to analyse the possible relationship with meteorological factors.	*Aedes aegypti* and *Ae. albopictus*	Identification of areas with different levels of risk for arboviruses based on 3 years of sampling. The findings permitted the identification of priority areas for vector control, reflecting distinct epidemiological and entomological scenarios that require the differential planning of vector control programs.	[139]

2020	Nascimento et al.	South/PR	English	Study aspects of the oviposition of the vector, compare ovitraps with larval surveys to determine the number of sentinel ovitraps necessary per home, evaluate the ideal number of pallets per trap for the reliable determination of *Ae. aegypti* infestation, and to identify the best location for the installation of ovitraps in homes for surveillance and control.	*Aedes aegypti*	The data obtained from the ovitraps were more sensitive for detecting the presence or abundance of *Ae. aegypti*, and indicated that two traps at each surveillance point are sufficient for conventional monitoring practices.	[140]
2020	Pedrosa et al.	Southeast /MG	English	Investigate the potential influence of rising winter temperatures as a critical factor for the local invasion of *Aedes*.	*Aedes aegypti* and *Ae. albopictus*	The results reinforce concerns that climate change will probably promote an increase in the availability of favorable habitats for the mosquito species and, consequently, the transmission of arboviruses.	[141]
2020	Sá et al.	Southeast /MG	English	Monitor the seasonality of *Ae. aegypti* using ovitraps and correlate the data with a dengue notification in the municipality in 2018.	*Aedes aegypti*	Ovitraps with beer yeast solution were effective not only for the monitoring of *Ae. aegypti*, but also the detection of other mosquitoes, such as *Culex*. The significant amounts of eggs and larvae, principally of *Ae. aegypti*, detected in ovitraps emphasize the need for intense monitoring and preventive measures to combat the mosquito, which may help reduce the indigence of dengue fever.	[142]
2020	Santos et al.	Northeast/ PE	English	Verify the association between a set of meteorological variables (precipitation, temperature, humidity, wind velocity and solar radiation) and the oviposition dynamics of *Ae. aegypti*.	*Aedes aegypti*	Precipitation affects *Aedes* oviposition dynamics in the study area. The narrow range of the other meteorological variables, in particular, temperature and humidity make the area ideal for the reproduction of *Aedes*, supporting continuous reproduction throughout the year and, in turn, the constant transmission of arboviruses.	[143]

2020	Soares et al.	North/PA	English	Evaluate the distribution of *Ae. albopictus* and its preference for either natural or artificial oviposition sites.	*Aedes albopictus*	*Aedes albopictus* was detected, raising epidemiological concerns given the role of the species in the transmission of arboviruses. The mosquito has adapted rapidly to urban environments.	[144]
2021	Macedo et al.	Northeast /CE	English	Analysis of the costs of arboviruses to government and households, the cost-effectiveness of interventions, the chronicity of chikungunya, and the acceptance, sustainability, and governance of vector control actions.	*Aedes aegypti*	Mobilising the community, government, professionals, and scholars to combat *Ae. aegypti* will be essential to reduce the social and economic impacts of viruses such as dengue, chikungunya, and Zika.	[145]
2021	Martinez et al.	Central west/GO	English	Test the efficacy of a simple dissemination device in combination with different granular formulations of IP 46 against *Ae. aegypti* adults under laboratory, semi-field, and field conditions.	*Aedes aegypti*	The findings of the study strongly support efforts to develop mycoinsecticides against mosquito vectors.	[146]
2021	Oliveira et al.	Northeast/ MA	English	Verify the efficiency of ovitraps combined with the biolarvicidal bacteria *Saccharopolyspora spinosa* and *Bacillus thuringiensis* for the monitoring of *Ae. aegypti* and *Ae. albopictus* eggs under laboratory and field conditions.	*Aedes aegypti* and *Ae. albopictus*	Ovitraps combined with the two biolarvicides provide a viable alternative for the monitoring of *Ae. aegypti* and *Ae. albopictus*, as confirmed by the high oviposition rates of the two species under laboratory conditions. Trapping efficiency was also noted under field conditions, given that the traps were positive for mosquito eggs in all combinations. The two biolarvicides are clearly efficient at attracting both mosquitoes, which facilitates the use of ovitraps as a monitoring tool.	[147]
2021	Da Silva et al.	North/AM	English	Evaluate the efficiency of different oviposition traps and the capture of *Ae. aegypti* and *Ae. albopictus* adults under field conditions in the South and	*Aedes aegypti* and *Ae. albopictus*	Trap efficiency appeared to have been enhanced by the grass infusion, which is known to attract *Aedes*, in comparison with plain water. The Vectobac WG (*Bacillus thuringiensis israelensis* - BTI) biolarvicide used here (as well as other	[148]

				North regions of Brazil to validate new effective and economical tools for vector monitoring.		BTI-based products) may also be an important contribution to monitoring, given the proven efficacy of BTI for the control of *Aedes* larvae.	
2021	Teixeira et al.	Northeast/ BA	English	Find evidence of the simultaneous infection *Ae. aegypti* larvae with two or more viral species to confirm the existence of the vertical co-transmission of arboviruses.	*Aedes aegypti*	First evidence of the simultaneous infection of *Ae. aegypti* larvae by two different arboviruses. The concomitant infection of *Ae. aegypti* by DENV, ZIKV, and CHIKV in a single region was also observed. The LIR*Aa* method proved effective for predicting the circulation of the different arboviruses in the vector.	[149]
2022	Sanchez-Gentriz et al.	Northeast/ RN	English	Understand the dynamics of the incidence of dengue in Natal over a four-year period, and develop models to forecast dengue infection rates based on *Ae. aegypti* capture rates.	*Aedes aegypti*	The incidence of dengue was associated with the socioeconomic status of the neighborhood. The ovitrap data correlated closely with the incidence of dengue, and provide a better predictor of outbreaks, given the potential for earlier detection (4–6 weeks in advance) in comparison with the number of dengue cases (one week).	[150]
2022	Nascimento et al.	South/PR	English	Evaluate the influence of climatic factors, including temperature, precipitation, relative humidity, wind speed, and atmospheric pressure, on the oviposition behavior of mosquitoes using mathematical models.	*Aedes aegypti*	The regression model determined that temperature was the only variable that influenced the number of *Ae. aegypti* eggs, with an increase of 1°C expected to result in a 54.03% increase in the number of eggs deposited. The greater incidence of dengue during periods with higher temperatures reflects this tendency. This pattern is intrinsically related to the global warming predicted by the IPCC, which may contribute to greater densities of the vector.	[151]
2022	Pereira et al.	Southeast /MG	English and portuguese	Verify whether ovitraps are effective for the capture of *Ae. aegypti* eggs and the relationship between captures and the reduction	*Aedes aegypti*	The number of cases of dengue recorded in 2019 was 20% lower than in 2016, despite the ongoing increase in the population of the municipality, while the number of eggs collected in the	[152]

				in the number of probable cases of dengue between 2019 and 2021.		ovitraps increased from 2019 to 2021, with an OPI of over 40% in most of the cycles analyzed.	
2022	Piovezan et al.	Southeast/ SP	English	Evaluation of the effectiveness of entomological surveillance and control measures for *Ae. aegypti.*	*Aedes aegypti*	Suppressing breeding sites is one of the most difficult and costly means of controlling *Ae. aegypti* populations, given the need to remove a significant proportion of potential habitats.	[153]
2022	Souza et al.	South/PR	English	Determine the spatial and temporal distribution of *Ae. aegypti* and *Ae. albopictus* and evaluate the influence of climatic variables on these species in the area of a recent dengue outbreak.	*Aedes aegypti* and *Ae. albopictus*	*Aedes albopictus* was the predominant species until 2014. The first autochthonous cases of DENV and *Ae. aegypti* had already been detected in the neighboring town of Paranaguá in the same period. The introduction of *Ae. aegypti* was likely facilitated by the highway that connects Antonina to Paranaguá, passing through Morretes. Tourism, the town's principal economic activity contributed to the influx of people and transportation.	[154]

[1] State abbreviations: AM – Amazonas, AP – Amapá, BA – Bahia, CE – Ceará, DF – Distrito Federal, ES – Espírito Santo, GO - Goiás, MA – Maranhão, MG – Minas Gerais, MS – Mato Grosso do Sul, MT – Mato Grosso, PA – Pará, PE – Pernambuco, PI – Piauí, PR – Paraná, RJ – Rio de Janeiro, RN – Rio Grande do Norte, RR – Roraima, SP – São Paulo, SE – Sergipe, TO – Tocantins.
